# Single-Anastomosis Duodenal Jejunal Bypass Improve Glucose Metabolism by Regulating Gut Microbiota and Short-Chain Fatty Acids in Goto-Kakisaki Rats

**DOI:** 10.3389/fmicb.2020.00273

**Published:** 2020-02-21

**Authors:** Xiang Yu, Zhuangwei Wu, Zhigao Song, Hongbin Zhang, Junfang Zhan, Hao Yu, Hongyan Huang, Baolin Yang, Lang Xie, Xiaojiang Dai, Weiguo Zhao, Jinlong Yu, Liangping Wu

**Affiliations:** ^1^Department of General Surgery, Zhujiang Hospital, Southern Medical University, Guangzhou, China; ^2^Center for Translational Medicine, The First Affiliated Hospital, Sun Yat-sen University, Guangzhou, China; ^3^Department of Medical Experimental, Guangzhou General Hospital of Guangzhou Military Command, Guangzhou, China; ^4^Health Management Center, Guangzhou First People’s Hospital, School of Medicine, South China University of Technology, Guangzhou, China; ^5^Department of Metabolic Surgery, UDM Medical Group, Guangzhou, China; ^6^Department of Metabolic Surgery, Jinshazhou Hospital of Guangzhou University of Traditional Chinese Medicine, Guangzhou, China

**Keywords:** single-anastomosis duodenal jejunal bypass, gut microbiota, short-chain fatty acids, glucose metabolism, type 2 diabetes

## Abstract

In recent years, bariatric surgery has emerged as a promising treatment for type 2 diabetes. Bariatric surgery is known to cause alterations in the relative abundance and composition of gut microbiota, which may lead to alterations in the levels of Short-Chain Fatty Acids (SCFAs) that are produced during fermentation by gut microbes. However, little is known about the mechanism of improved glucose metabolism mediated by gut microbiota following bariatric surgery. The aim of our study was to explore whether changes in gut microbiota and in fecal SCFA could be detected following single-anastomosis duodenal jejunal bypass (DJB-sa) surgery, a type of bariatric surgery, and whether these alterations might be related to the improvement of glucose metabolism. To this end, we performed DJB-sa or SHAM surgery on Goto-Kakisaki (GK) rats. We then compared the glucose metabolism as well as changes in gut microbiota and SCFAs levels between both groups. Our results showed that DJB-sa surgery was associated with a significant decrease in fasting blood glucose (FBG), intraperitoneal glucose tolerance test (IPGTT), and fasting serum insulin (FSI). And, DJB-sa led to a change in the composition of gut microbiota including an increase in the relative abundance of SCFA-producing bacteria (*Bifidobacterium* and *Subdoligranulum*). Moreover, the levels of six SCFAs in feces, as well as the intestinal expression of SCFA receptors including G-protein-coupled receptor 41 (GPR41), G-protein-coupled receptor 43 (GPR43), and G-protein-coupled receptor 109A (GPR109A), and the expression of Glucagon-like peptide-1 (GLP-1) displayed a significant increase following DJB-sa compared with the Sham group. Thus, the gut microbiota may contribute to the improvement of glucose metabolism in type 2 diabetes following DJB-sa. In conclusion, our study shows that DJB-sa improves glucose metabolism by modulating gut microbiota and by increasing short-chain fatty acid production.

## Introduction

Globally, diabetes and its complications have reached epidemic levels, which has caused an enormous threat to human health and the social economy. It has been estimated that 451 million adults worldwide were suffering from diabetes mellitus in 2017 (Zheng et al., 2018). Bariatric surgery has emerged as an effective treatment of type 2 diabetes (T2DM) and the amount of patients undergoing bariatric procedures has been steadily increasing during the past few years (Zhong et al., 2016). The rational explanation for this success has been provided by a number of long-term clinical research studies which have shown that bariatric surgery might help the type 2 diabetic patients manage their disease better by improving the state of the average glucose level and by lowering the complication rate (Mingrone et al., 2015).

Over the past decades, many studies have suggested that bariatric surgery can improve glucose metabolism and insulin sensitivity in T2DM. The improvement may depend on several potential mechanisms, which mainly include altered gut hormones, reduced hepatic and pancreatic triglycerides, altered bile acid, altered neural signaling, and reprograming of intestinal glucose metabolism (Batterham and Cummings, 2016). Recently, several studies focused on the relationship between improved glucose metabolism and changes in the gut microbiota upon bariatric surgery. Bariatric surgery was found to cause changes in *Firmicutes, Bacteroidetes, Proteobacteria*, and *Actinobacteria* at the phylum level (Palleja et al., 2016; Murphy et al., 2017). However, few studies were designed to investigate the changes in gut microbiota upon Duodenal-jejunal bypass (DJB) surgery. DJB, a type of bariatric surgery sparing the stomach, resulted in an improvement of glucose metabolism. Single-anastomosis duodenal jejunal bypass (DJB-sa) is similar to DJB but with a simplified reconstruction method (Torres, 2019). Moreover, DJB-sa did not alter the biliopancreatic diversion or accelerate biliopancreatic flow to the lower intestine (Han et al., 2015; Weng et al., 2017). Although it has been reported that the new biliopancreatic diversion might be an effective mechanism to improve the condition of T2DM after DJB (Dubourg et al., 2013; Weng et al., 2017), DJB-sa did not display this effect. Therefore, this surgical procedure will be a better choice to investigate the effect of gut microbiota on glucose metabolism following the surgical procedure.

Gut microbiota mainly include *Firmicutes, Bacteroidetes, Proteobacteria*, and *Actinobacteria* at the phylum level (Gaboriau-Routhiau et al., 2011; Fujio-Vejar et al., 2017), of which *Firmicutes and Bacteroidetes* are the dominant species and account for ∼90% of the population (Ndeh and Gilbert, 2018). They convert food ingredients into various nutrients that humans are able to utilize to sustain life (Barratt et al., 2017). Intestinal microbiota produce short-chain fatty acids (SCFAs) *in vivo* mainly by way of bacterial fermentation of carbohydrates (Zhao et al., 2018; Chen et al., 2019). SCFAs mainly include acetate, propionate, butyrate, isobutyrate, valerate, and isovalerate and the lack of these SCFAs has been associated with T2DM (Canfora et al., 2015; Zhao et al., 2018).

Thus, in order to have a better understanding of the relationship between bariatric surgery, gut microbiota, and SCFA, we studied changes in the gut microbiota and SCFA following DJB-sa surgery in Goto-Kakisaki (GK) rats and determined whether these changes were associated with improved glucose metabolism. By applying this model of diabetes, the potential mechanisms by which DJB-sa is beneficial to T2DM patients can be explained from a new perspective (Arora and Bäckhed, 2016; Zhang et al., 2016).

## Materials and Methods

### Animals

Thirteen-week-old male Goto-Kakizaki (GK) rats were purchased from the Laboratory Animal Center of Cavens (Nanking, China). The rats were separately housed in individually ventilated cages under constant temperature (22–24°C) and constant humidity (55–65%) in a 12-h light/dark cycle. All rats were given free access to tap water and fed with standard food (14% calories from fat) in the Southern Medical University Laboratory Animal Center. Random blood glucose was measured with a glucometer (Roche, Mannheim, Germany) from tail veins. Three out of fifteen rats were excluded since their blood glucose values did not exceed 11.1 mmol/L. The 12 rats with random blood glucose levels ≥ 11.1 mmol/l were randomly divided into a DJB-sa group (*n* = 6) and a SHAM-surgery group (*n* = 6). The Animal Care and Use Committee of Guangzhou General Hospital of Guangzhou Military Command approved all animal procedures.

### Surgical Procedures

Before surgery, the rats were fasted for 12 h and anesthetized by intraperitoneal injection of amobarbital sodium (50 mg/kg).

#### DJB-sa

**FIGURE 1:**
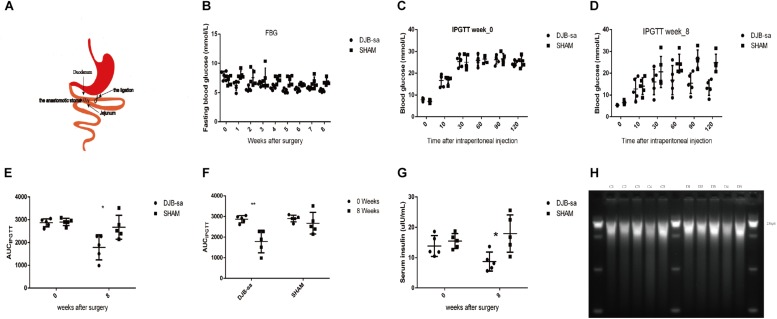
**(A)** Anatomical drawing of the DJB-sa procedure. The anastomotic stoma is the anastomosis of duodenum and jejunum. **(B)** FBG. **(C)** IPGTT at week 0. **(D)** IPGTT at week 8. **(E)** The significant difference of AUC_IPGTT_ between two groups prior to and 8 weeks post-surgery. **(F)** The significant difference of AUC_IPGTT_ between 0 and 8 weeks in each group. **(G)** Significant differences in FSI between two groups before and 8 weeks after surgery. **(H)** Agarose electrophoresis of total DNA from gut microbiota. The length of the total DNA was about 21 kb and showed no signs of degradation. C1–C5: Total DNA from feces of the DJB-sa group 8 weeks post-surgery; D1–D5: Total DNA from feces of the SHAM group 8 weeks post-surgery. Differences between the DJB-sa group and the SHAM group were assessed by the paired Student’s *t*-test, *p < 0.05, **p < 0.01.

After disinfection, a midline abdominal incision was performed (about 3–5 cm) and the duodenum was exposed and located at 1 cm distal from the pylorus. Then, the duodenum was anastomosed (approximately 0.8 cm) to the jejunum at 20 cm distal to the ligament of Treitz in a side-to-side manner using a 6-0 silk suture (Ningbo Medical Needle, China). The distal duodenum was closed with a 5-0 silk suture near the anastomosis. DJB-sa surgery does not change the diversion of biliopancreatic flow and expedites it to the distal ileum (Figure 1A).

#### SHAM Surgery

Following the midline abdominal incision, the duodenum was exposed at 1 cm distal to the pylorus and the jejunum was exposed at 20 cm distal to the ligament of Treitz. The two intestinal segments were subjected to the same ∼0.8 cm incision and then sutured with a 6-0 silk suture.

On the day of the surgery, the rats were only allowed to have water. For 3 days post-surgery the rats were fed a 0.9% saline solution including 5% glucose. Subsequently, they were fed the same standard chow throughout the remaining experimental period. All rats were sacrificed after 12 h fasting at week 8. No antibiotics were used.

### Detection of Blood Glucose and Serum Insulin

After fasting for 12 h, fasting blood glucose (FBG) was measured in all rats prior to surgery and every week post-surgery. The intraperitoneal glucose tolerance test (IPGTT) was conducted prior to and 8 weeks post-surgery. After 12 h of fasting, the rats were administrated 20% of glucose (2 g/kg) by intraperitoneal injection. Blood glucose was measured at 0, 10, 30, 60, 90, and 120 min following the injection. Prior to and 8 weeks after surgery, blood samples were acquired through the orbital venous sinus. The blood samples were collected into 2 mL Eppendorf tubes and centrifuged at 4°C for 10 min at 1100 × *g*. The plasma supernatant was transferred to a fresh 2 mL Eppendorf tube and stored at −80°C. Serum insulin was detected with an ELISA Kit (Ray Biotech, United States).

### Intestinal Tissues and Feces Collection

The intestinal tissues were harvested 8 weeks post-surgery. Feces were collected from living rats prior to DJB-sa procedures and again 8 weeks postoperatively. Fecal stools were placed directly into 2 mL sterile, nuclease-free microtubes at the time of defecation. Fecal samples were stored at −80°C after liquid nitrogen flash freezing.

### DNA Isolation and Agarose Gel Electrophoresis

DNA was isolated from the stool samples (0.5 g) by using the FastDNA SPIN Kit for Feces (MP Biomedicals, United States) in a FastPrep Instrument (MP Biomedicals), according to the manufacturer’s instructions. The DNA samples were stored at −80°C until needed for DNA electrophoresis and 16S rDNA Microbiome Analysis.

The DNA sample (2 μl) was added to 5 μl loading buffer (MP Biomedicals) and separated by standard 1.5% agarose (BIOWEST, Spain) gel electrophoresis at 100 V for 30 min at room temperature. The DNA marker was λDNA/Hind III (DongSeng Biotech, China). The running buffer was Tris-acetate EDTA (TAE) (Leagene, China). The gel was stained with Gold View and visualized under UV light.

### 16S rRNA Microbiome Analysis

The fecal microbiota profile was analyzed by 16S rRNA gene sequencing. The sequencing (V3-V4 region) was performed with the Illumina MiSeq platform (2 × 250 bp) according to a standard protocol in Huayin Medical Laboratory Center Co., Ltd. (Guangzhou, China). The 16S rRNA sequencing data generated were used to analyze taxonomic classification and relative abundance by using the software suites including VSEARCH (v2.3.4), R (v3.3.2) and Quantitative Insight Into Microbial Ecology (QIIME) (v1.80). The methods to analyze the Operational Taxonomic Units (OTU) included OTU Venn Graph, Principal Component Analysis, OTU Rank Curve, and species accumulation curves. The analyses of relative abundance included bacterial profiling, and construction of a heatmap, and genus specific phylogenetic tree. Total pairs read number (20 samples clean reads) was 2,077,215. Species diversity analysis of each sample was performed with Alpha diversity analysis (including observed species, Chao, Ace, Shannon and Simpson) to estimate fecal microbial species richness and species diversity. Beta-diversity was calculated with Bray-Curtis, weighted UniFrac and unweighted UniFrac. In addition, the beta diversity was analyzed by heatmap, principal coordinates analysis (PCoA) and, weighted_unifrac according to the Unweighted Pair Group Method with Arithmetic mean (UPGMA). Linear discriminant analysis (LDA) effect size (LEfSe) was used to analyze significant differences between two groups and evaluate the effect size of each gut bacteria. The Wilcox test and MetaStats were used to analyze the significant differences of relative abundance between two groups or before and after surgery. *P*-value < 0.05 was considered significant).

### Sample Preparation and Analysis of SCFAs in Feces

Feces samples (1 g) were thawed at room temperature and transferred to a 2 ml sterile Eppendorf tube. Water (1 mL) was added, and the sample was vortexed for 5 min and then mixed ultrasonically for 15 min. Then, the mixtures were centrifuged for 10 min at 15000 rpm in a refrigerated high-speed centrifuge. The supernatant was transferred to a 4 ml tube. Next, anhydrous sodium sulfate (0.5 g), 10 μL of 50% sulfuric acid, and 2 ml diethyl ether were added. The mixture was vortexed for 2 min and centrifuged at 5000 rpm for 15 min. Finally, the supernatant (1 ml) was removed and collected into a glass autosampler vial.

The fecal SCFAs were determined and analyzed by using a Gas Chromatography-Mass Spectrometer (GC-MS) system. The GC-MS analysis was performed using a TRACE1300 gas chromatograph equipped with an ISQ Single Quadrupole Mass Spectrometer (Thermo Scientific) with a DB-FFAP capillary column (30 m × 0.25 mm × 0.25 μm film thickness) (Agilent Technologies) and AI1300 auto-sampler (Thermo Scientific). 1 μl of supernatant sample was injected into the inlet with a 3 min of solvent delay time and 1:5 split mode. The flow rate of nitrogen (purity of 99.999%) carrier gas was kept at 1 mL/min. The injection port, ion source and quadrupole were at 200, 250, and 230°C, respectively. The initial oven temperature was 90°C for 1 min, and was then increased to 200°C at the rate of 12°C/min, and finally kept at this temperature for 2 min and 20 s. The selected ion (m/z) values to determine each SCFA including acetic, propionic, isobutyric, butyric, isovaleric, and valeric acids were 43, 45, 57, 60, 73, 74, and 87, respectively. Hydrogen, air, and nitrogen served as composition gasses and their flow rates were 30, 300, and 30 mL/min, respectively. The total run-time for each analysis was 15 min. Data were analyzed by the Xcalibur Qual Browser (Thermo Scientific). The external standard method was used to calculate the contents of each SCFA.

### Western Blotting

The intestinal tissues were washed with cold PBS and lysed with lysis buffer (Beyotime, China). The protein quantity was detected using the BCA protein assay Kit (Beyotime, China). Proteins were separated on 10% sodium dodecyl sulfate-polyacrylamide (SDS-PAGE) gels and transferred to polyvinylidene difluoride (PVDF) membranes (Millipore, Germany). Then, the membranes were blocked with 5% BSA in Tris-buffered saline containing 0.2% Tween-20 (TBST) for 2 h at room temperature, washed extensively with TBST and incubated with the primary antibody: rabbit anti-GPR41 (Cohesion, United Kingdom), rabbit anti-GPR109A (Cohesion, United Kingdom), mouse anti-GLP-1 antibody (Abcam, United States), rabbit anti-GAPDH antibody (Proteintech Group, United States), mouse anti-tubulin antibody (Ray Antibody Biotech, China) at 4°C overnight. Finally, the membranes were incubated with secondary antibody: goat anti-rabbit IgG Secondary Antibody (Sigma, United States) or goat anti-mouse IgG Secondary Antibody (Invitrogen, United States) and the blots were developed with a chemiluminescence reagent (Millipore, Germany).

### Immunohistochemistry

Paraffin-embedded intestinal tissues were cut into 4-μm-thickness sections, heated at 65°C for 1 h, dewaxed with xylene for three 10-min cycles, blocked with 3% of H_2_O_2_ and treated with Citrate Antigen Retrieval Solution (Beyotime, China) to retrieve the antigen at 90–95°C for 15 min. The slides were blocked with normal goat serum at 90–95°C for 30 min and incubated with the primary antibody: rabbit anti-GPR43 antibody (Bioss, China) overnight at 4°C. Then the slides were incubated with the secondary antibody (Dako, United States) for 30 min at 37°C and stained with diaminobenzidine (DAB) (Dako, United States). Next, the sections were counterstained with hematoxylin and cleared with xylene. Six random fields near the intestinal mucosa were captured with a microscope and analyzed using Image Pro Plus6.0 (Lu et al., 2006; Wang Tilz et al., 2006).

### Immunofluorescence Microscopy

Sections were prepared as described above for immunohistochemistry. The sections were heated, dewaxed with xylene, rehydrated using an alcohol gradient and washed with PBST. The slides were treated with Citrate-EDTA Antigen Retrieval Solution (BOSTER, China) at 90–95°C for 15 min. Then, the sections were penetrated with 0.5% Triton X-100 (Solarbio, China) at room temperature for 20 min, blocked with 5% normal goat serum at 37°C for 1 h and incubated with the primary antibody: rabbit anti-GPR41 antibody, rabbit Anti-GPR43 antibody, rabbit Anti-GPR109A antibody, or mouse Anti-GLP-1 antibody at 4°C overnight. Next, the slides were treated with the secondary antibody: Anti-rabbit IgG (H + L), F(ab’)2 Fragment (Alexa Fluor^®^488 Conjugate) (Cell Signaling Technology, United States), or anti-mouse IgG (H + L), F(ab’)2 Fragment (Alexa Fluor^®^555 Conjugate) (Cell Signaling Technology, United States) at 37°C for 50 min and mounted with DAPI (Beyotime, China). The slides were captured by fluorescence microscopy and analyzed using ImageJ software.

### Statistical Methods

All data are presented as mean values ± SD. Area under the curve (AUC) for IPGTT was calculated by trapezoidal integration. All analyses were performed with SPSS 19.0 software. Statistically significant differences were detected with unpaired *t*-tests or Wilcoxon-test. A *P*-value of <0.05 was considered statistically significant. All tables and graphs were analyzed and constructed using GraphPad Prism v 6.0 software.

## Results

### Changes in Blood Glucose and Serum Insulin After DJB-sa

In both the DJB-sa group and the SHAM group, 5 rats remained alive 8 weeks after the surgery. The levels of FBG (Figure 1B), intraperitoneally glucose tolerance test (IPGTT) (Figures 1C–F) and fasting serum insulin (FSI) (Figure 1G) had decreased and glucose metabolism had improved significantly at 8 weeks following surgery in the DJB-sa group compared with the SHAM group.

### DJB-sa Surgery Resulted in Changes of Gut Microbiota

In order to investigate the effect of DJB-sa surgery on the gut microbiota, 16SrRNA sequencing analysis (V3 through V4) was performed with the total DNA extracted from the fecal samples. The DNA used for the sequencing analysis did not display any signs of degradation (Figure 1H).

**FIGURE 2:**
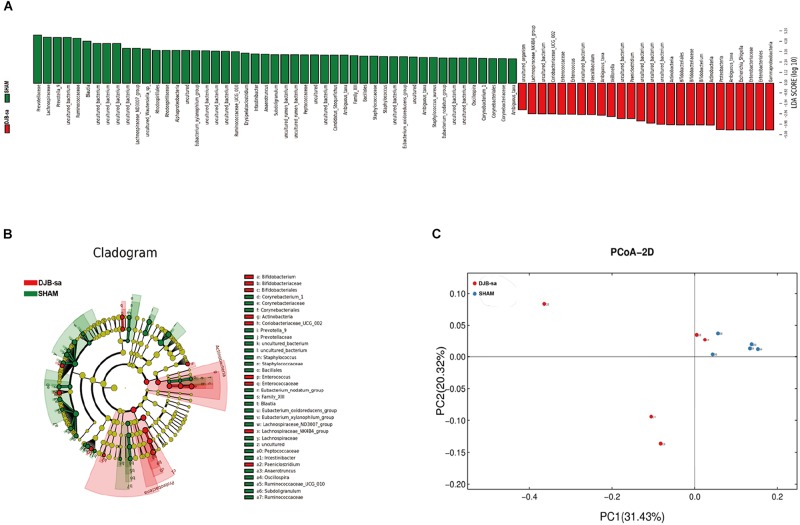
Linear discriminant analysis effect size (LEfSe). **(A)** The Histogram represents the relative abundances at phylum, class, order and family levels between the DJB-sa group and the SHAM group at 8 weeks post-surgery. **(B)** The Cladogram of plots presented the LEfSe results of the biological structure of the gut microbiome. Gut bacteria marked with small circles highlight significant differences of relative abundance between the two groups. **(C)** Principal coordinate analysis (PCoA) based on weighted Unifrac metrics indicating the different beta diversity of gut microbiota of the fecal samples in the two groups.

The linear discriminant analysis effect size (LEfSe) illustrated the relative abundances at the phylum, class, order and family levels (Figure 2). The histogram revealed significant differences in the gut microbiota between the DJB-sa group and the SHAM group at 8 weeks post-surgery. The green bars represented a higher relative abundance of gut bacteria in the SHAM group and the red bars indicated an enrichment of bacteria in DJB-sa group (Figure 2A). The results of the Cladogram revealed the relative abundances to be the same as those shown in the Histogram. Gut bacteria marked with small circles highlight significant differences in relative abundance between the two groups. The circles have different layers, which represent different taxonomic ranks (phylum, class, order, family, and genus, respectively) from inside to outside. The diameter of each circle is proportional to the abundance of the taxon. Each node (circle) represents a taxon; the green nodes represent the enrichment of gut bacteria at 8 weeks post-surgery in the SHAM group; the red nodes indicate a higher relative abundance in the DJB-sa group. Yellow nodes account for those gut bacteria that were not statistically and biologically different in terms of abundance between the two groups (Figure 2B). The two diagrams identified the clades of gut bacteria and explained systematically the greatest differences between the DJB group and SHAM group (Figures 2A,B). We used principal coordinate analysis (PCoA) to visualize the differences in the fecal samples between the two groups. The red points each represent a fecal sample at 8 weeks post-surgery in the DJB group and the blue points indicate the samples from the SHAM group. The distance between the points represents the approximate differences between gut bacterial communities in the fecal samples (Figure 2C). The results revealed that the relative abundance and composition of the gut microbiota differed between the two groups.

**FIGURE 3:**
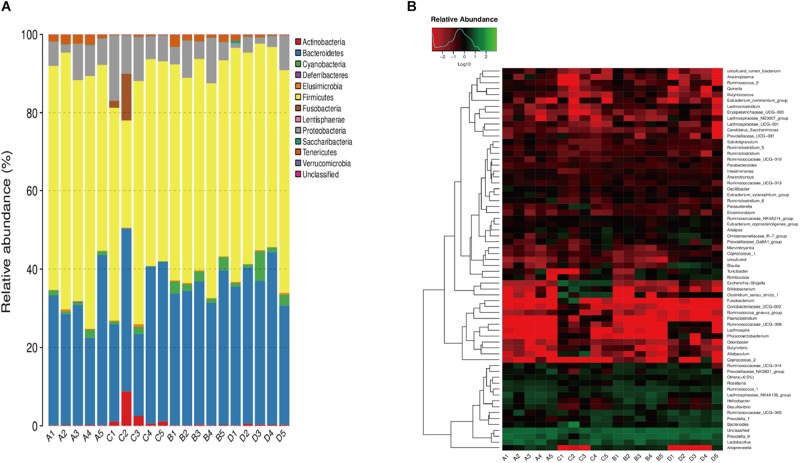
A1–A5: DJB-sa group prior to surgery; B1–B5: SHAM group prior to surgery; C1–C5: DJB-sa group 8 weeks post-surgery; D1–D5: SHAM group 8 weeks post-surgery. **(A)** Relative abundance of the most abundant OTUs at the phylum level in fecal samples of GK rats. **(B)** Heatmap of the most abundant OTUs at the genus level in fecal samples. The color of the small rectangles in the graph represents the relative abundance of each genus in each sample. The quantity of relative abundance is indicated by the variation of color from blue to red. Blue represents a reduction and red indicates an increase.

We compared the relative abundance of gut bacterial operational taxonomic units between the DJB-sa group and the SHAM group. The taxon analysis revealed statistically significant differences in the composition of gut microbiota following DJB-sa surgery compared with the SHAM group (Figure 3). At the phylum level, the most abundant species belonged to the *Actinobacteria*, *Bacteroidetes*, *Firmicutes*, and *Proteobacteria*. The DJB-sa surgery was associated with a significant increase of *Actinobacteria* (*p* < 0.01) and *Proteobacteria* (*p* < 0.05) (Figure 3A). Meanwhile, at the order level, the DJB-sa surgery resulted in the distinct increase of species from *Bifidobacteriales* (0.79%) and *Enterobacteriales* (0.79%) and the reduction of species from *Bacillales* (1.59%), *Corynebacteriales* (2.54%) and *Rhodospirillales* (3.17%) (Table 1 and Supplementary Table S1), as analyzed with the Wilcoxon-test (*p* < 0.05). Nineteen genera were significantly different after DJB-sa surgery (Table 2 and Supplementary Table S2), as analyzed using the Wilcoxon-test (*p* < 0.05). In the DJB-sa group, the most abundant species were *Bifidobacterium* and *Escherichia_Shigella*. Compared with the SHAM group, the increased genera included *Bifidobacterium* (0.79%), *Enterococcus* (2.12%), *Faecalibaculum* (0.79%), *Escherichia_Shigella* (0.79%), *Intestinibacter* (3.61%), *Coriobacteriaceae_UCG_002* (0.75%), *Lachnospiraceae_NK4B4_group* (3.61%) and *Paeniclostridium* (1.78%) and the decreased genera were *Anaerotruncus* (3.17%), *Candidatus_Stoquefichus* (1.78%), *Corynebacterium_1* (2.54%), *Eubacterium_oxidoreducens_group* (0.79%), *Erysipelatoclostridium* (1.59%), *Eubacterium_xylanophilum_ group* (3.17%), *Lachnospiraceae_ND3007_group* (3.17%), *Oscillospira* (4.49%), *Ruminococcaceae_UCG_010* (3.17%), *Staphylococcus* (1.59%), *Subdoligranulum* (3.17%) (Table 2 and Figure 3B).

**TABLE 1:** Statistically significant differences in relative abundance of gut microbiota at the order level between the DJB-sa group and the SHAM group (8 weeks post-surgery).

Relative abundance (%)	DJB-sa Group	SHAM Group	*p*-value
*Bacillales*	0.002956 ± 0.002514	0.088033 ± 0.106483	*
*Bifidobacteriales*	2.363922 ± 3.120338	0.033388 ± 0.0486	**
*Corynebacteriales*	0 ± 0	0.023665 ± 0.032864	*
*Enterobacteriales*	7.175186 ± 4.075694	0.131782 ± 0.202218	**
*Rhodospirillales*	0.18729 ± 0.10942	0.506787 ± 0.222747	*

*P < 0.05, **P < 0.01.

**TABLE 2:** Statistically significant differences in relative abundance of gut microbiota at the genus level between the DJB-sa group and the SHAM group (8 weeks post-surgery).

Relative abundance (%)	DJB-sa Group	SHAM Group	*p*-value
*Anaerotruncus*	0.268088 ± 0.103935	0.429496 ± 0.095664	*
*Bifidobacterium*	2.36345 ± 3.12068	0.031067 ± 0.044802	**
*Candidatus_Stoquefichus*	0.002748 ± 0.006144	0.103781 ± 0.142999	*
*Coriobacteriaceae_UCG_002*	0.171566 ± 0.225374	0 ± 0	**
*Corynebacterium_1*	0 ± 0	0.022368 ± 0.033062	*
*Enterococcus*	0.210766 ± 0.164387	0.015741 ± 0.02641	*
*Erysipelatoclostridium*	0.048005 ± 0.045587	0.237916 ± 0.098302	*
*Escherichia_Shigella*	7.170672 ± 4.076723	0.128414 ± 0.203286	**
*Eubacterium_oxidoreducens_group*	0.031736 ± 0.016755	0.075911 ± 0.031803	**
*Eubacterium_xylanophilum_group*	0.276446 ± 0.132526	0.652306 ± 0.166386	*
*Faecalibaculum*	0.239382 ± 0.158532	0.019082 ± 0.008937	**
*Intestinibacter*	0.003625 ± 0.004127	0.012658 ± 0.005435	*
*Lachnospiraceae_ND3007_group*	0.107005 ± 0.165109	0.811791 ± 0.774009	*
*Lachnospiraceae_NK4B4_group*	0.195915 ± 0.189627	0.021653 ± 0.042321	*
*Oscillospira*	0.000471 ± 0.001054	0.03277 ± 0.04081	*
*Paeniclostridium*	0.501956 ± 0.394156	0.000589 ± 0.001316	*
*Ruminococcaceae_UCG_010*	0.140191 ± 0.076948	0.401195 ± 0.263155	*
*Staphylococcus*	0.002956 ± 0.002514	0.075949 ± 0.092133	*
*Subdoligranulum*	0.087656 ± 0.054237	0.212553 ± 0.0728	*

*P < 0.05, **P < 0.01.

### Changes in the Levels of Fecal SCFAs Following DJB-sa Surgery

**FIGURE 4:**
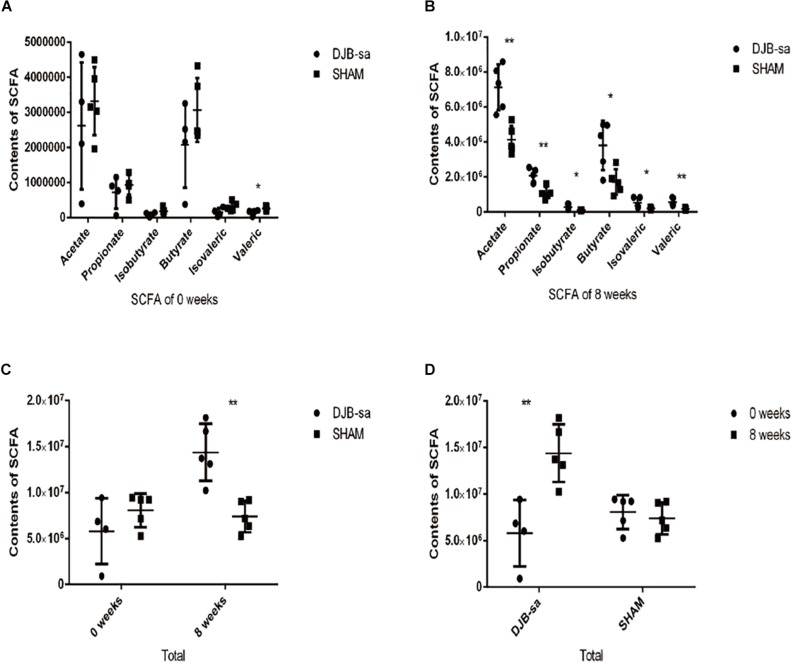
**(A)** Pre-surgery fecal content of various SCFAs in the DJB-sa and the SHAM groups. From left to right, the six SCFAs included acetate, propionate, isobutyrate, butyrate, isovalerate, and valerate. **(B)** Illustration of the statistically significant differences of each SCFA, 8 weeks after DJB-sa surgery. **(C)** The total represented the sum of the six SCFA contents. The total SCFA content had increased at 8 weeks in the DJB group compared with the SHAM group. **(D)** The total change of each group at 8 weeks compared with 0 weeks. Differences between the DJB group and SHAM group were assessed by the paired Student’s *t*-test, *p < 0.05, **p < 0.01.

The fecal SCFAs detected by GC-MS were acetate, propionate, isobutyrate, butyrate, isovalerate, and valerate. DJB-sa surgery significantly increased the contents of fecal SCFAs (*P* < 0.05). Moreover, each SCFA was significantly more abundant in the DJB-sa group compared with the SHAM group. One fecal sample from the before surgery DJB-sa group was discarded due to inadequate collection. The total SCFA content (the sum of six SCFAs) in the DJB-sa group 8 weeks post-surgery showed a significant increase compared with the SHAM group and the preoperative control (0 weeks) (Figure 4). The changes in SCFA levels were consistent with the modulation of gut microbiota caused by the surgery.

### Changes in the Intestinal Expression of Short Chain Fatty Acid Receptors

**FIGURE 5:**
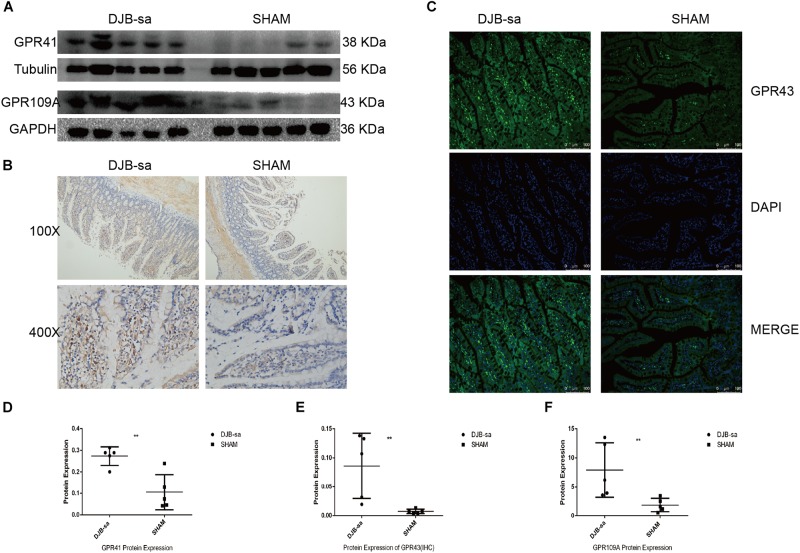
**(A)** The left five bars represent the expression of GPR41 and GPR109a in intestinal tissues from the DJB-sa group and the right five bars represent their expression in the SHAM group. **(B)** Change of GPR43 protein expression was assessed using immunohistochemistry. Magnification 100×, the top half of **(B)**; Magnification 400×, the bottom half of (B), Brown areas represent positive staining of GPR43. **(C)** Changes in GPR43 protein expression in the intestines assessed using immunofluorescence. Magnification 200x. The cell nucleus was stained blue with DAPI. Green spots indicate the positive fluorescence staining of GPR43. **(D)** GPR41 expression was increased in the intestines detected by western blotting. **(E)** Statistically significant increase of GPR43 expression in intestinal tissues in the DJB-sa group as detected by immunohistochemistry. **(F)** GPR109A expression was increased in the intestines as detected by western blotting. Differences between the DJB-sa group and SHAM group were assessed by the paired Student’s *t*-test, **p < 0.01.

We next investigated the effect of the DJB-sa surgery on the expression of SCFA receptors. Protein extracted from the intestinal tissue was used to investigate the change in G-protein-coupled receptor 41 (GPR41) expression. Our results showed that the GPR41 expression was significantly increased at 8 weeks following DJB-sa (Figures 5A,D). And, we observed that G-protein-coupled receptor 43 (GPR43) levels in intestinal tissues had increased significantly after the DJB-sa surgery (Figures 5B,C,E). Moreover, the G-protein-coupled receptor 109A (GPR109A) had markedly increased as well (Figures 5A,F).

### The Co-expression of Short Chain Fatty Acid Receptors and GLP-1

**FIGURE 6:**
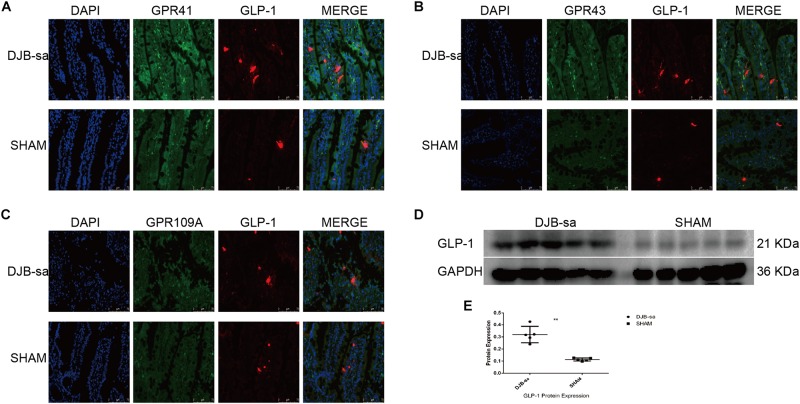
**(A)** The co-expression of GPR41 and GLP-1 in intestinal tissues. The nuclei were stained with DAPI (blue spots). The green spots represent positive expression of GPR41. The red spots indicate the positive fluorescence staining of GLP-1. Magnification 400x. **(B)** The co-expression of GPR43 (green spots) and GLP-1 (red spots). Magnification 400x. **(C)** The co-expression of GPR109A (green spots) and GLP-1 (red spots). Magnification 400x. **(D)** Differences in expression of GLP-1 following DJB-sa compared with SHAM surgery. The left five bars represent the expression of GLP-1 in intestinal tissues from the DJB-sa group and the right five bars represent its expression in the SHAM group. **(E)** The GLP-1 expression was increased in the intestines as detected by western blotting. Differences between the DJB-sa group and SHAM group were assessed by the paired Student’s *t*-test, **p < 0.01.

In order to investigate the effect of the SCFA receptors on the secretion of Glucagon-like peptide-1 (GLP-1), we detected the relationship by using immunofluorescence co-localization. We observed that the green spots (representing the SCFA receptors: GPR41, GPR43, and GPR109A) were located close to the red spots (representing the GLP-1) (Figures 6A–C). In addition, the GLP-1 expression in intestinal tissues had markedly increased at 8 weeks after DJB-sa (Figures 6D,E).

## Discussion

In our study, we performed DJB-sa and SHAM surgery on GK rats. The DJB-sa surgery resulted in levels of FBG, IGTT and FSI that had decreased significantly at 8 weeks after DJB-sa. We observed that the composition and abundance of intestinal microbiota were effectively altered and that the fecal contents of six SCFAs were significantly increased after DJB-sa surgery. Moreover, we proved that DJB-sa surgery resulted in the increased expression of SCFAs receptors and GLP-1 in the intestinal tissues.

As reflected by the results of FBG, IPGTT and FSI tests, we suggested that the DJB-sa procedure led to a significant improvement of the glucose metabolism of T2DM. These findings were consistent with previous studies indicating that bariatric surgery could be a treatment for T2DM (Song et al., 2018; Hofsø et al., 2019; Yu et al., 2019). Although it has been shown that DJB surgery improved glucose metabolism and insulin secretion by suppressing the glucose uptake by the downregulation of glucose transporters, activating the insulin signaling pathway, and inhibiting the inflammatory signals which induced beta cell apoptosis (Jurowich et al., 2013; Wu et al., 2018; Li et al., 2020), but, a recent study indicated that the new biliopancreatic diversion (NBPD) was a main contributing factor to the glucose-lowering effects of DJB compared with simple duodenal-jejunal exclusion and SHAM surgery in T2DM (Weng et al., 2017). However, the DJB-sa procedure we performed achieved the same hypoglycemic effect without the NBPD. Therefore, there must be some other mechanism responsible for these observations.

As shown by the results of LEfSe, taxon analysis, and principal coordinate analysis, we observed that the gut microbiota underwent significant structural changes including community composition and relative abundance. In our study, two species increased significantly at the phylum level and 19 genera were altered at the genus level. At the phylum, the *Actinobacteria and Proteobacteria* had a significant increase while the *Bacteroidetes* and *Firmicutes* had not noticeably altered 8 weeks following DJB-sa. However, it has been reported that DJB led to an alteration in the composition of gut microbiota mainly by increasing the relative abundance of *Firmicutes* and *Proteobacteria* and by decreasing the relative abundance of *Bacteroidetes* at the phylum (Zhong et al., 2016). Although the alteration at the phylum level that we observed was not the same as that reported by others, our results may suggest that the effective improvement on T2DM caused by DJB-sa surgery is associated with a small proportion of gut microbiota and not with the dominant species (*Firmicutes* and *Bacteroidetes)*. At the genus level, 19 genera had a significant change and six of them might be associated with T2DM. Four increased genera were *Bifidobacterium*, *Faecalibaculum*, *Escherichia_Shigella*, *Enterococcus* and two decreased genera were *Intestinibacter* and *Oscillospira*. *Bifidobacterium* was found to be decreased in T2DM patients, but bariatric surgery resulted in an increased relative abundance of *Bifidobacterium*, *Faecalibaculum* and *Enterococcus* (Miyachi et al., 2016; Kim et al., 2017; Guo et al., 2018; Lee et al., 2019). Moreover, recent studies also found that T2DM patients undergoing metformin treatment showed an increased abundance of *Escherichia_Shigella* along with a reduced population of *Intestinibacter* (Bryrup et al., 2019) while a higher relative abundance of *Oscillospira* was positively associated with the pathogenesis of diabetes (Wu et al., 2019). The changes in six genera were the same as reported in previous studies on drug and surgery treatment of T2DM, but it is the first time that bariatric surgery is observed to cause alterations in the relative abundance of *Escherichia_Shigella*, *Intestinibacter*, and *Oscillospira*. Although recent studies suggested that bariatric surgery led to obvious alterations in the gut microbiota with improved glucose metabolism in T2DM, the mechanism remained to be validated.

In T2DM, the altered gut microbiota potentially affect the fecal metabolome, the immune status and the intestinal redox status and inflammation (Lopez-de-Andres et al., 2018; Piccolo et al., 2018; Wang et al., 2018; Tian et al., 2019). T2DM was reportedly affected by the decrease of butyrate-producing species in rats (Li et al., 2019; Zhou et al., 2019). In our study, we observed that levels of *Bifidobacterium* and *Faecalibaculum* had significantly increased after DJB-sa. Additionally, they have been identified as important commensal bacteria that can produce SCFAs by fermenting fiber (Zhao et al., 2018; Liu et al., 2019). SCFAs have been shown to improve glucose homeostasis and the deficiency is associated with T2DM (Canfora et al., 2015). Contrary to the changes of SCFAs in T2DM, we observed that six SCFAs displayed a significant increase following DJB-sa surgery. SCFAs, as a kind of nutrient source, can improve glucose metabolism by binding to the receptors (GPR43, GPR41 and GP9109A) (Kim et al., 2018; Priyadarshini et al., 2018; Xu et al., 2018). Moreover, we also observed that the expression of SCFA receptors (GPR43, GPR41 and GPR109A) and GLP-1 significantly increased at 8 weeks after DJB-sa surgery. It has been shown that GPR41 can be activated by propionate and butyrate and that GPR43 can be triggered by acetate and propionate (Tolhurst et al., 2012). This combination causes the release of glucagon-like peptide-1 (GLP-1) and peptide YY (PYY) (Sanna et al., 2019), which in turn stimulate insulin secretion (Zhao et al., 2018). Although few studies have reported that SCFA receptor levels had increased after bariatric surgery, the change in GLP-1 was the same as found in previous studies (Nauck and Meier, 2016). GLP-1 is mainly produced by L cells, which is located in the intestinal. GLP-1 could inhibit gastric emptying, suppress glucagon secretion, stimulate insulin secretion and enhance the β-cell proliferation, all of which contributed to lowering the blood glucose in T2MD (Kim and Egan, 2008). And, from our results, the blood glucose level was obviously decreased. Therefore, with the increased SCFAs, the receptors and GLP-1, we could infer that the SCFAs/SCFA receptors (GPR41, GPR43 and GPR109A)/GLP-1 signaling pathway might contribute to the improvement of glucose metabolism in T2MD rats performed with DJB-sa surgery (Lee et al., 2018; Bolognini et al., 2019).

However, there are some limitations to our study. While DJB-sa surgery has been shown to result in significant alterations in the structure and relative abundance of gut microbiota, the exact mechanism remain unclear. Although we could not ensure that the improvement of glucose metabolism was directly caused by the increased production of SCFAs, we can infer that the alteration of SCFA might be a valuable contributing factor. As for the increased or decreased gut species, especially *Bifidobacterium*, our results provide more evidence for the need to develop and apply microecologics to treat T2DM. In view of the increased fecal contents of SCFA, our results introduce the new and important idea that SCFAs would affect the glucose metabolism in T2DM following bariatric surgery. It might be possible to improve the condition of T2DM by fortifying common foods with SCFAs.

## Conclusion

Our results indicate that DJB-sa surgery led to an improved glucose metabolism and an increase in the relative abundance of SCFA-producing species. Moreover, our results also show a significant increase of six SCFAs as well as an increase in the expression of SCFA receptors and of GLP-1 in intestinal tissues following DJB-sa. Thus, we inferred that the increase in SCFAs might play a positive role to improve the glucose metabolism in association with the activation of the SCFA receptor (GPR41, GPR43 and GPR109A)/GLP-1 signaling pathway.

## Data Availability Statement

The datasets generated for this study can be found in the DDBJ/ENA/GenBank under the accession KDPS00000000. The version described in this paper is the first version, KDPS01000000. The individual sequences were available under the accession numbers KDPS01000001–KDPS01697213.

## Ethics Statement

The animal experiments in this study were approved by the Animal Care and Use Committee of Guangzhou General Hospital of Guangzhou Military Command.

## Author Contributions

JY and LW conceived and designed this study. XY and ZW performed this research. HZ and ZS provided their technical assistance. XY wrote the manuscript. All authors have commented on, and approved the manuscript.

## Conflict of Interest

The authors declare that the research was conducted in the absence of any commercial or financial relationships that could be construed as a potential conflict of interest.

## Supplementary Material

TABLE S1 The gut microbiota at the order level between the DJB-sa group and the SHAM group (8 weeks post-surgery).

TABLE S2 The gut microbiota at the genus level between the DJB-sa group and the SHAM group (8 weeks post-surgery).
